# A CT-FFR-guided unroofing procedure for repairing the anomalous origin of the left coronary artery—a case report

**DOI:** 10.3389/fcvm.2023.1167698

**Published:** 2023-05-22

**Authors:** Hairun Zuo, Chengyi Xu, Li Wang, Chengwei Liu, Li Liu, Xi Su

**Affiliations:** Department of Cardiology, Wuhan Asia Heart Hospital, Wuhan, China

**Keywords:** anomalous aortic origin of a coronary artery, sudden cardiac death, computed tomography angiography, fractional flow reserve, unroofing procedure

## Abstract

Anomalous aortic origin of a coronary artery (AAOCA) is a congenital malformation of the coronary arteries that includes several subtypes. It is a leading cause of sudden cardiac death in young people, especially in competitive athletes. An accurate diagnosis and identification of high-risk patients with AAOCA for referral for surgical repair can help in the management of these patients. However, current diagnostic tools such as invasive angiography, echocardiography, and intravascular ultrasound have known limitations in visualizing coronary orifices and characterizing vessels. In this case report, we report on a 14-year-old adolescent who suffered from repeated incidents of syncope during exercise. Using the computed tomographic fractional flow reserve (CT-FFR) technique, we diagnosed AAOCA, which revealed that his left coronary artery (LCA) originated from the right sinus of Valsalva and ran between the aorta and the pulmonary artery with an intra-arterial wall course (∼20 mm in length), with an abnormal FFR of the LCA at rest. The patient was referred for undergoing unroofing surgery, and the results of repeat CT-FFR showed a significantly improved FFR of the LCA. The patient resumed his normal physical activities without the recurrence of syncope. In this report, we highlight the usefulness of CT-FFR as a non-invasive, feasible, and effective tool to guide whether a patient with AAOCA requires surgical revascularization and to evaluate the effectiveness of the procedure after surgery.

## Introduction

Anomalous aortic origin of a coronary artery (AAOCA) refers to the anomaly of the left or right coronary artery originating from the inappropriate aortic sinus and can be classified into several types. Although there is increasing recognition of this congenital disorder due to the development of cardiac imaging techniques, the true incidence in the general population remains unknown. It has been reported that AAOCA is one of the leading causes of sudden cardiac death (SCD) (1–3), with anomalous origin of the left coronary artery being associated with a higher risk of SCD, particularly in competitive athletes (1–6). Therefore, identifying high-risk patients with AAOCA and adopting appropriate management, such as surgical correction, is of the utmost importance. In this case report, we report a case of AAOCA in an adolescent who complained of recurrent episodes of syncope during exercise (playing basketball and running). Coronary computed tomography angiography (CTA) revealed that the left coronary artery (LCA) originated from the left side of the right sinus of Valsalva and ran between the aorta and the pulmonary artery with an intra-arterial wall course of approximately 20 mm in length. We then used a novel technique, called computed tomographic fractional flow reserve (CT-FFR) to assess the functional status of the coronary arteries. The results showed that the FFR of the LCA was lower than the normal value. The patient underwent a modified unroofing procedure after a multidisciplinary discussion. Postprocedural CT-FFR revealed a normal range of FFR, and the patient resumed his normal activities without recurrence of syncope. In this report, we highlight the usefulness of CT-FFR as a non-invasive, feasible, and effective tool to guide whether a patient with AAOCA requires surgical revascularization and evaluate the effectiveness of the procedure in the post-surgical period.

## Case presentation

**Figure 1:**
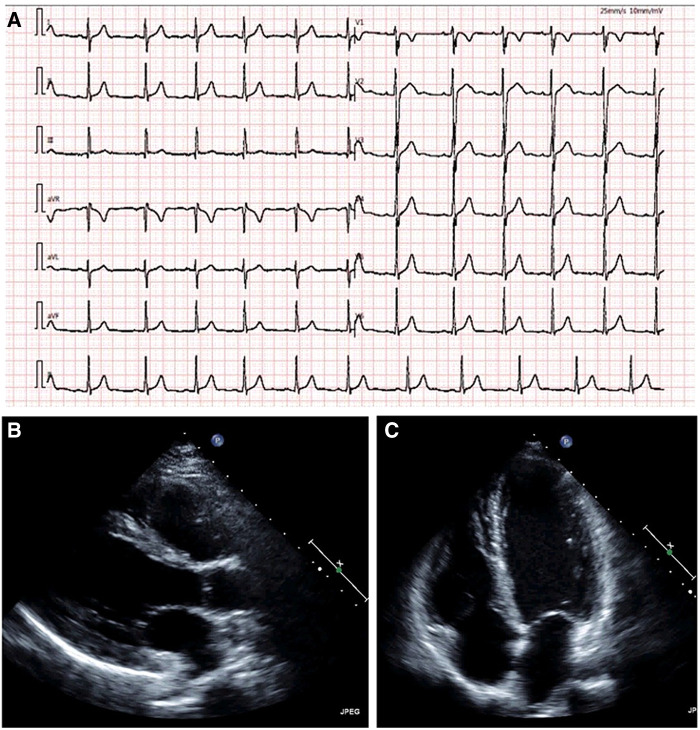
Admission electrocardiogram shows sinus arrhythmia without significant abnormalities. (**A**) Echocardiography shows normal cardiac structure and function (**B, C**). (**B**) parasternal long-axis view; and (**C**) apical four-chamber view.

**Figure 2:**
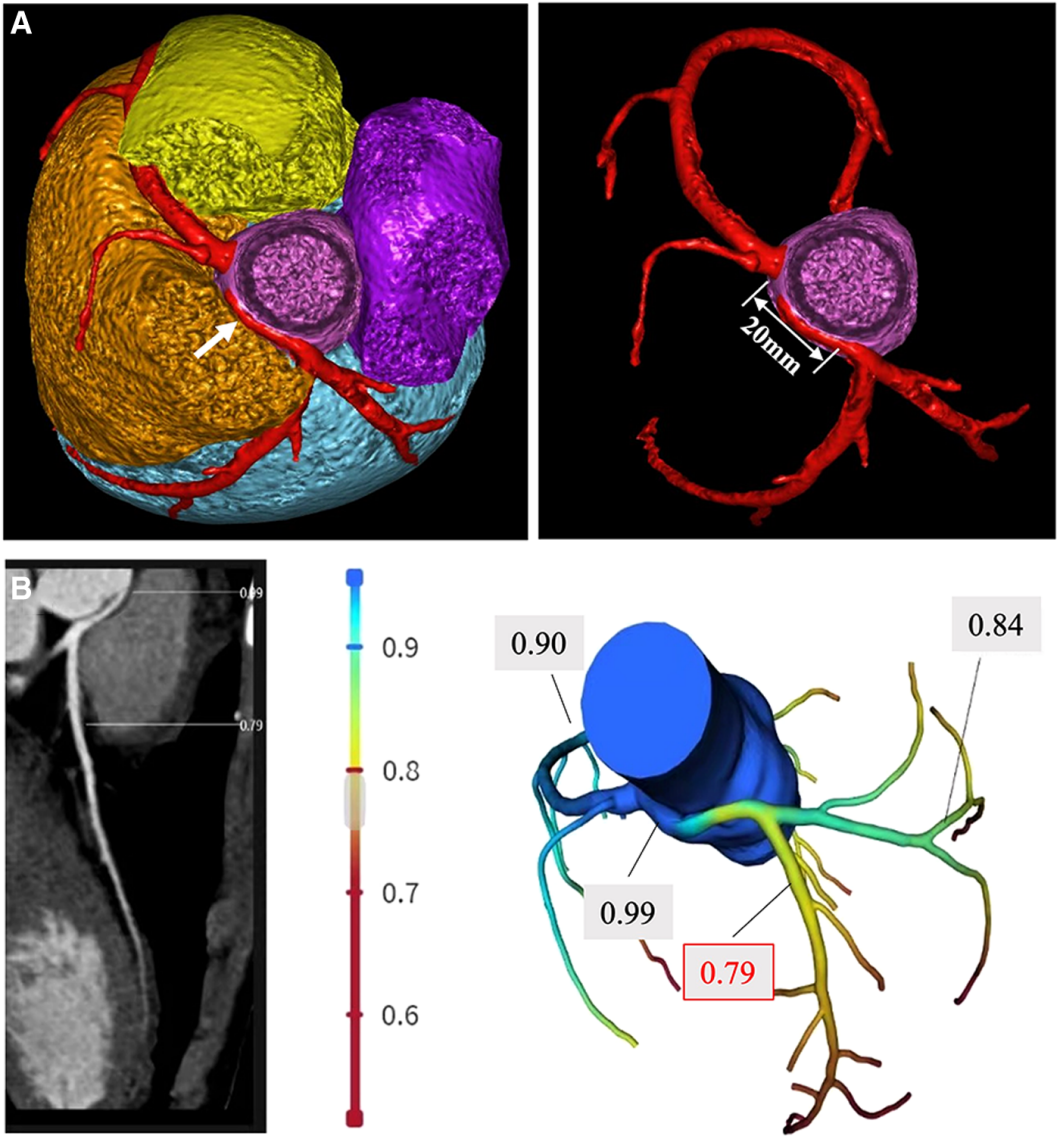
Preoperative CT-FFR measurement. (**A**) Coronary CTA reveals the left coronary artery originating from the right sinus of Valsalva and running between the aorta and the pulmonary artery (white arrow), the length of the intra-arterial wall course is approximately 20 mm, and the origin of the right coronary artery is normal. (**B**) CT-FFR measurement shows a reduced FFR of the left coronary artery (LCA, 0.79) and a normal FFR of the left circumflex artery (LCX, 0.84) and the right coronary artery (RCA, 0.90).

**Figure 3:**
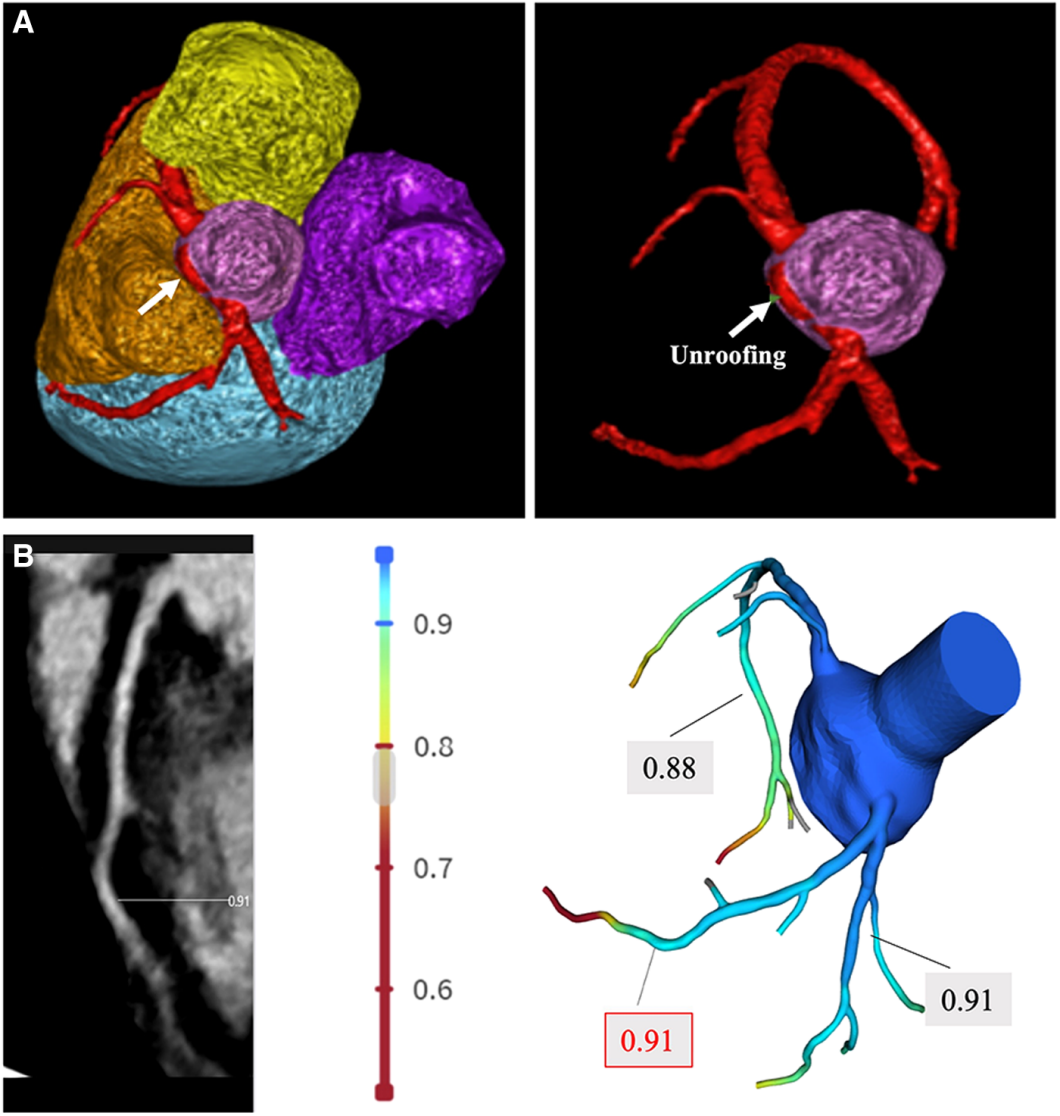
Post-procedure CT-FFR measurement. (**A**) Coronary CTA shows revascularization of the LCA. The intramural segment of the coronary artery of the LCA is unroofed (white arrow) and corrected to originate from the left sinus of Valsalva. (**B**) CT-FFR technique was performed 1 week after the revascularization procedure, and the result showed a significantly improved FFR of the LCA (0.91) and normal range FFR of the LCX (0.91) and RCA (0.88).

A 14-year-old adolescent presented to our hospital with a complaint of syncope during exercise (playing basketball). He reported feelings of dizziness, sweating, and blackness before fainting. He denied having chest pain or abnormalities in limb movement before and after the occurrence of the episodes. He suffered from two similar episodes in the preceding 2 years while playing basketball and while running. He denied having a family history of coronary artery disease, cardiomyopathy, hypertension, and diabetes. He was diagnosed as experiencing a vagal syncope because of a positive tilt test result a year ago in a local hospital. However, a review of his previous medical records did not reveal any abnormalities. A physical examination on admission revealed normal levels of blood pressure (120/60 mmHg), heart rate (78 beats/min), respiratory rate (18/min), and oxygen saturation of 99% (room air). Cardiac and pulmonary examinations showed normal heart sounds without murmurs and with clear lungs. Laboratory test results indicated normal levels of blood glucose, high-sensitivity troponin, CK-MB, N-terminal B-type natriuretic peptide (NT-proBNP), D-dimer, CBC w/diff, and CMP. The renal and hepatic functions were within normal limits. The baseline ECG displayed sinus rhythm (HR = 78 bpm) without significant abnormalities (Figure 1A). Transthoracic echocardiography (TTE) showed normal cardiac structure and function (Figure 1B). To further investigate the possible reason for syncope, coronary CTA was performed, which revealed an anomalous origin of the LCA originating from the left side of the right sinus of Valsalva and ran between the aorta and the pulmonary artery with an intra-arterial wall course of approximately 20 mm in length (Figure 2A). To clarify whether the anomalous origin of the LCA was the real reason for syncope, we performed CT-FFR on all coronary branches at rest, which revealed an abnormal FFR of the left anterior descending artery (LAD, 0.79) but a roughly normal FFR of the left circumflex artery (LCX, 0.84) and the right coronary artery (RCA, 0.90) (Figure 2B). CT-FFR is a physiologic simulation technique that models coronary flow from routine coronary CTA. To evaluate lesion-specific ischemia, CT-FFR is measured 2 cm distal to a stenotic lesion. A CT-FFR value >0.8 is considered normal, values between 0.76 and 0.8 denote a borderline condition, and a value of 0.75 or less represents an abnormal condition. Since CT-FFR of the LAD was lower than normal at rest, we reasoned that it should be even lower during physical exercise. The cardiac team concluded that the AAOCA was the reason behind the patient’s syncope, following which a surgical correction with a modified unroofing procedure was performed. One week after surgery, repeat CT-FFR was performed to evaluate the effectiveness of the corrective surgery, and the result showed a successful revascularization of the LCA (Figure 3A) and a significantly improved LAD flow with CT-FFR 0.91 (Figure 3B). CT-FFR of the LCX (0.91) and RCA (0.88) remained within normal limits. The patient was discharged 10 days after surgery and followed up on an outpatient basis. He soon resumed his normal activities and had no recurrence of syncope.

## Discussion

AAOCA is a congenital malformation of the coronary artery and is one of the leading causes of SCD in young athletes (4–6). It involves the left main coronary artery arising from the right sinus, the right coronary artery originating from the left sinus, or both coronary arteries originating from the same sinus (4, 6). Anatomically, there are five routes of an anomalous coronary artery, namely, interarterial, subpulmonic, pre-pulmonic, retro aortic, and retrocardiac routes (7, 8). The clinical manifestations of AAOCA mainly depend on the course of the anomalous coronary artery and whether it is associated with an intra-arterial wall course. It is believed that the abnormal RCA originating from the left sinus, which is 6–10 times more common than the anomalous origin of the LCA from the right sinus, is a benign lesion and rarely causes clinical symptoms (3, 7, 9). However, an anomalous origin of the LCA from the right sinus, when running between the aorta and the pulmonary artery, combined with an intra-arterial wall course, is more likely to cause exercise-induced syncope and even SCD (3, 9). Possible mechanisms of SCD include proximal coronary hypoplasia, dynamic ostial narrowing, intussusception of the intimal wall of the intramural segment, and interarterial compression during exercise (10). The exact pathophysiological mechanism of SCD in patients with AAOCA remains unclear, and it may be secondary to acute ischemia during or after exercise that triggers ventricular tachyarrhythmias and fibrillation (11). Therefore, efforts to make an accurate diagnosis, identify high-risk patients, and provide treatment in a timely manner are of the utmost importance.

TTE is an excellent diagnostic technique used to evaluate patients with suspected or known cardiac disease, as it is non-invasive, convenient, and economical. However, it has limited accuracy in detecting the coronary ostia and intramural course of the anomalous coronary artery origin. Successful diagnosis of AAOCA with TTE depends on experienced surgeons, optimal image quality, in-depth knowledge of AAOCA, and satisfactory spatial resolution of the equipment. A study by Lorber et al. (12) showed very poor agreement between institutional and expert TTE reports and surgical findings of AAOCA measures (i.e., interarterial course, intramural course, and acute angle takeoff). Transesophageal echocardiography (TEE) can provide more detailed information and clear images of the coronary ostia and has been used to identify AAOCA (12–14); however, it is not a routine tool for imaging AAOCA. Although invasive coronary angiography (ICA) is considered the gold standard for detecting coronary artery disease, it is not a reasonable choice for the diagnosis of AAOCA because of its invasive nature, relatively high cost, and limitations in characterizing AAOCA vessels. ICA usually cannot visualize the course of the coronary artery running through the aortic wall, and sometimes, it is hard to locate the coronary orifice of abnormal origin (15). In a registry study of 23,300 ICA cases, the original course of anomalous coronary arteries was not identified in 41% of patients (16).

Nowadays, an excellent diagnostic technique called CT-FFR, which combines imaging display and functional evaluation of coronary artery stenosis, is increasingly used in patients with coronary artery disease as a gatekeeper to the catheterization laboratory. In this case report, Ruixin-FFR (Shenzhen Ruixin Intelligent Medical Technology Co., Ltd., China) is used for CT-FFR calculation. Ruixin-FFR obtains the coronary CTA image file through a data communication interface. Based on the artificial intelligence image processing algorithm, the centerline and outline of the three-dimensional coronary artery tree can be extracted, and the accurate three-dimensional modeling of the coronary artery tree can be performed to obtain its three-dimensional geometric information. This can be combined with the quantification of physiological parameters to obtain the boundary conditions of hemodynamics. Based on the simulation and analysis of hydrodynamics, and combined with the above three-dimensional model and boundary conditions, the FFR of each position on the coronary tree is calculated. Several studies (17–19) demonstrated that the diagnostic performance of CT-FFR was superior to coronary CT angiography alone for the detection of hemodynamically relevant coronary stenoses, which significantly reduced the number of false-positive findings detected by coronary CTA. CT-FFR was particularly useful in the diagnosis of AAOCA: CTA could clearly display the abnormal origin, the intramural course of the coronary artery, and the angle of the coronary orifice by three-dimensional reconstruction. FFR could be an adjunct to determine the functional significance of coronary stenosis (20). Unlike invasive FFR, CT-FFR is an image postprocessing technique that applies computational fluid dynamics to coronary CTA image data to simulate the physiological function of the coronary artery. It uses standardized coronary CTA images to evaluate the hemodynamic differences of coronary artery stenosis without additional CT scanning, radiation dose, or drug use and can therefore provide FFR values non-invasively. This technique is particularly suitable for young patients because of its non-invasiveness, lower radiation exposure compared to ICA, and lower risk of complications compared to ICA. For AAOCA, this technique is more suitable due to the potential difficulties associated with invasive testing. Lee et al. (21) examined 37 consecutive adult patients with invasive FFR, and they noted that one patient developed an ostial anomalous right coronary artery (ARCA) dissection during ICA, and placement of a guiding catheter was not possible in two patients. In our patient, CTA clearly revealed the anomalous origin of the left coronary artery and its running course between the aorta and the pulmonary artery with an intra-arterial wall course of approximately 20 mm in length. CT-FFR showed a lower-than-normal value of the LAD (0.79) at rest. A CT-FFR value >0.8 is considered normal, values between 0.76 and 0.8 are considered borderline, and a value of ≤0.75 is regarded as abnormal. We hypothesized that the FFR value could be even lower during exercise, thereby inducing myocardial ischemia, resulting in ventricular arrhythmias or low cardiac output, which could be the cause of the patient's symptom (recurrent syncope).

According to the guidelines (22), patients with AAOCA were recommended to restrict their activity and undergo surgical correction if they had symptoms of ischemic chest pain, or syncope suspected to be due to ventricular arrhythmias, or a history of aborted sudden cardiac death. Specifically, for patients with anomalous LCA originating from the right coronary sinus with an intra-arterial course, guidelines (22) recommend that these patients receive surgical correction as soon as possible, regardless of clinical symptoms. For asymptomatic individuals with AAOCA, or patients with AAOCA and a non-interarterial course subtype (i.e., pre-pulmonic, subpulmonic, retro-aortic, retro-cardiac), a conservative approach may be appropriate (22, 23). If the decision is made to pursue surgical repair, a modified unroofing procedure is usually preferred. Coronary artery bypass grafting is not recommended in the absence of concomitant obstructive coronary artery disease because of the potential for competitive flow from native vessels to cause graft failure. In our study, the patient received the unroofing procedure, repeated CT-FFR showed a significantly enlarged valve in the LAD (0.91) after surgery, and no recurrence of syncope during exercise was recorded at the 6-month follow-up. This may confirm that repeated exercise-induced syncope before surgery is associated with coronary artery ischemia during exercise.

In conclusion, AAOCA is a congenital malformation of the coronary arteries and one of the leading causes of SCD in adolescents and young adults. Efforts to identify high-risk patients and refer them for surgery can help save their lives. CT-FFR is a feasible and cost-effective technique that is particularly suitable for young patients due to its non-invasiveness, lower radiation exposure compared to CA, and lower risk of complication compared to CA. It can also be used to evaluate the effectiveness of surgical correction, and it is superior to TTE and TEE in terms of diagnosis, risk stratification of AAOCA, and evaluation of surgical procedure efficacy. However, this study also has some limitations because of the following factors: the preoperative resting CT-FFR value is not sufficient to prove LAD ischemia, and we only put forward the case that CT-FFR has some guiding significance for the diagnosis and treatment of AAOCA patients. Further research is needed to confirm our findings.

## Data Availability

The original contributions presented in the study are included in the article, and further inquiries can be directed to the corresponding author.
